# *Histoplasma capsulatum* urinary antigen detection in a kidney transplant recipient with acute paracoccidioidomycosis: Case study and literature review

**DOI:** 10.1371/journal.pntd.0012472

**Published:** 2024-08-29

**Authors:** Matheus Oliveira Bastos, Andréa Gina Varon, Pedro Henrique Nascimento Theodoro, Eduardo Mastrangelo Marinho Falcão, Rosely Maria Zancopé-Oliveira, Antonio Carlos Francesconi do Valle, Rodrigo Almeida-Paes, Priscila Marques de Macedo

**Affiliations:** 1 Serviço de Atenção de Pacientes Internados, Instituto Nacional de Infectologia Evandro Chagas, Fundação Oswaldo Cruz (Fiocruz), Rio de Janeiro, Brazil; 2 Laboratório de Pesquisa Clínica em Dermatologia Infecciosa, Instituto Nacional de Infectologia Evandro Chagas, Fundação Oswaldo Cruz (Fiocruz), Rio de Janeiro, Brazil; 3 Laboratório de Micologia, Instituto Nacional de Infectologia Evandro Chagas, Fundação Oswaldo Cruz (Fiocruz), Rio de Janeiro, Brazil; Albert Einstein College of Medicine, UNITED STATES OF AMERICA

## Abstract

**Background:**

Paracoccidioidomycosis (PCM) and histoplasmosis are endemic fungal diseases in South America. Both can lead to lung involvement with fungal dissemination progressing to systemic and severe clinical manifestations, especially in immunosuppressed hosts. As the population of immunosuppressed individuals has been rising, a higher occurrence of fungal infections is predicted in this setting. This poses challenges regarding the differential diagnosis due to overlapping clinical and laboratorial findings, hampering the management of cases.

**Objectives:**

In this study, the authors discuss the occurrence of a false-positive *Histoplasma* urinary antigen detection in a kidney transplant recipient with acute PCM. Given the scarce information about this subject, a review on literature data is provided.

**Methods:**

A comprehensive literature search was conducted to investigate previous studies that found cross-reactivity between *Histoplasma* urinary antigen assays in human patients with confirmed diagnosis of PCM. Additionally, an update of PCM in transplant recipients is provided.

**Findings:**

The included studies reported 120 samples from patients with PCM tested for *Histoplasma* antigen, presenting an overall cross-reactivity of 51.67% and 17 cases of PCM in transplant recipients. CONCLUSIONS: The galactomannan urinary antigen developed to diagnose histoplasmosis can cross react with PCM, which may represent a concern in countries where both mycoses overlap.

## Author summary

Paracoccidioidomycosis (PCM) and histoplasmosis are severe fungal diseases occurring simultaneously in South America. They can cause similar clinical manifestations, especially in immunosuppressed hosts. This poses challenges regarding the differential diagnosis due to overlapping clinical and laboratorial findings, hampering the management of individual cases. In this study, the authors report the occurrence of a false-positive urinary test to detect histoplasmosis in a kidney transplant recipient with acute PCM. Given the scarce information about this subject, a review on literature data is provided. As conclusion, this study highlights the urinary test developed to diagnose histoplasmosis can cross react with PCM, which may represent a concern in countries where both mycoses overlap.

## Introduction

Paracoccidioidomycosis (PCM) and histoplasmosis are both endemic fungal diseases in South America. Their causative agents are the thermally dimorphic fungi *Paracoccidioides* spp. and *Histoplasma capsulatum*, respectively. Human infections occur through inhalation of conidia and other infective fungal particles present in the environment [1–3]. Although clinical manifestations are distinct, both infections can lead to lung involvement with fungal dissemination progressing to systemic and severe manifestations, especially in immunosuppressed hosts [4–6]. As the population of immunosuppressed individuals has been rising as a consequence of advances in treatment and long-term survival of transplant recipients, autoinflammatory and autoimmune disorders, and cancer therapy, a higher occurrence of endemic and opportunistic fungal infections in South America in this population is predicted [7,8]. This poses a significant challenge regarding the differential diagnosis due to overlapping clinical and laboratorial (mostly serological cross-reactions) findings, hampering the clinical management of individual cases.

Proper and early diagnosis of fungal infections is crucial to decrease mortality rates [9]. However, gold-standard techniques in routine practice are still histopathology and fungal culture of clinical specimens, which requires time, laboratorial infrastructure, supplies, and well-trained staff, scarcely available in some endemic regions [1,10]. Delays in diagnosis result in increased morbidity and mortality, especially in transplant recipients, who present impaired cellular adaptive immune response. In this context, molecular techniques and antigen detection are good strategies for a rapid and presumptive diagnosis of invasive fungal infections [9,11,12]. One of the main advances in histoplasmosis diagnosis was the introduction of *Histoplasma* antigen detection, generally performed in urine samples. The overall sensitivity of this test is 92% in transplant recipients, but specificity is limited due to cross-reactivity with other fungal infections such as aspergillosis, and endemic mycoses as blastomycosis, coccidioidomycosis, and talaromycosis which can result in inadequate diagnosis and clinical management [4,13–16].

*Paracoccidioides* spp. are primary systemic fungal pathogens rarely affecting transplant recipients [17]. However, changes in PCM epidemiology with increased cases in urban areas [18], as well as increasing numbers of solid organ transplants (SOT) in Brazil, mostly occurring in urban areas [19], can lead to a higher occurrence of this fungal disease in this population. A recent review of PCM in immunosuppressed patients described 10 cases of this mycosis in SOT recipients reported in the literature during the 30-year-period studied [6]. Although half of those patients were considered to have chronic PCM, all cases showed an opportunistic clinical pattern of this fungal disease, more severe, invasive, requiring hospitalization, and with high mortality rates. Proper diagnosis and management of PCM in SOT are not evidence-based and the high false-negative rates of antibody-based detection tests, as well as the numerous drug interactions between immunosuppressants used after transplantation and itraconazole, the first-line antifungal used to treat PCM, are challenging issues. Additionally, the lack of recommendation if a secondary prophylaxis is required after the end of PCM treatment in the context of a sustained immunosuppressive condition is a matter of concern.

In the present study, the authors report a case of acute PCM in a kidney transplant recipient who presented a false-positive *Histoplasma* urinary antigen. We also conducted a literature review on cross-reactivity between this diagnostic test and PCM, as well as an update on literature data about PCM in SOT recipients. Key issues regarding potential challenges in the management of these cases are further discussed.

## Methods

### Ethical statements

The Instituto Nacional de Infectologia Evandro Chagas Institutional Review Board approved this study (appreciation number 18524919.1.0000.5262). A written informed consent form was obtained from the participant of this study.

### Case study

**Fig 1:**
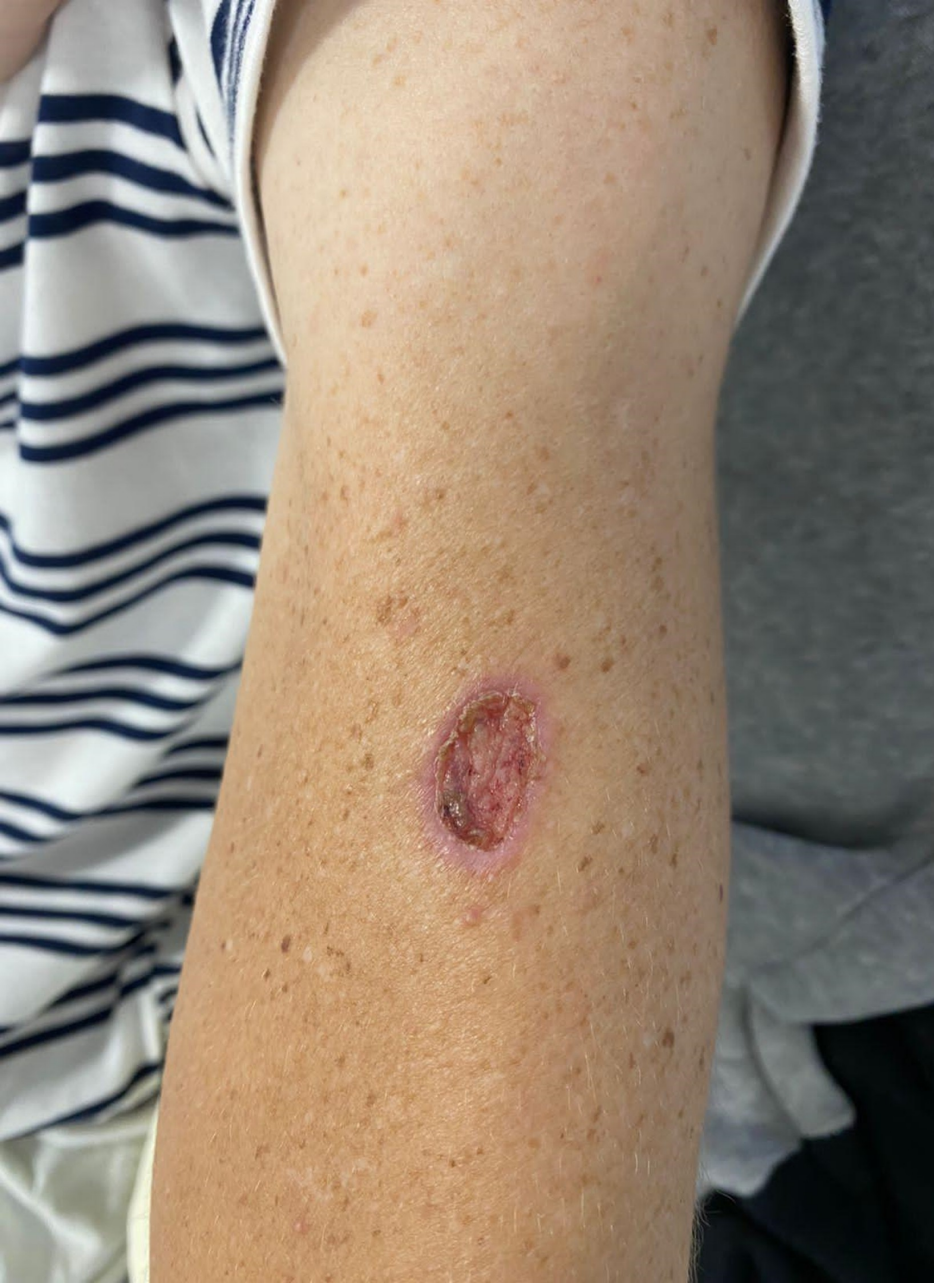
Flat ulcerated skin lesion in the left arm of the patient participating in this study.

A 42-year-old male patient from the urban area of Nova Friburgo municipality (mountainous region of Rio de Janeiro state, Brazil) first presented at our outpatient clinic in September 2022 with a history of multiple skin lesions evolving since 2021. Systemic symptoms consisted of low-grade daily fever, night sweats, weight loss, generalized lymphadenopathy, joint pain, dysphagia for solids, and hoarseness started in March 2022. He had a previous history of deceived-donor kidney transplant in 2016 due to a chronic kidney disease of unknown etiology and was under immunosuppression with tacrolimus 4 mg/day, sodium mycophenolate 720 mg/day, and prednisone 5 mg/day, reporting no history of rejection or opportunistic infections. He was living with other three healthy individuals in an apartment with an external area where they took care of plants. In addition, he reported no travels. He worked in a textile factory in the past and had a short-term job as taxi driver at the time of his first appointment in our institution. At admission, the patient presented diffuse lymph node enlargement in the cervical, supraclavicular, axillary, and inguinal chains. He also had single skin ulcers on the face, thorax, and left arm (Fig 1).

**Fig 2:**
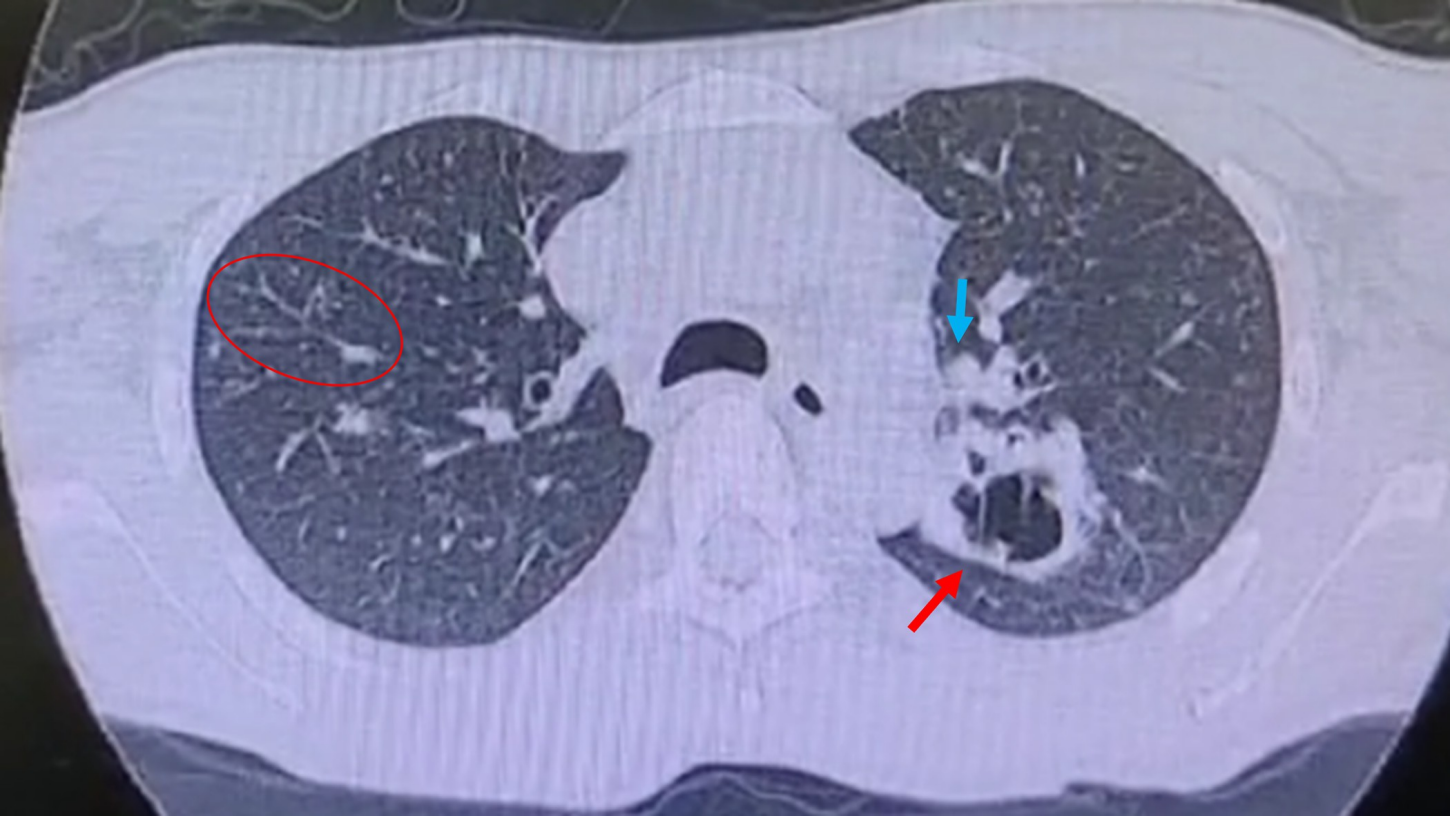
Computed tomography showing diffuse bilateral ground glass opacities, tree-in-bud sign (red circle), left upper lobe cavitation (red arrow), and mediastinal lymphadenopathy (blue arrow).

Otorhinolaryngology evaluation showed a fine-grained infiltration of aryepiglottic and arytenoid folds. Neurological evaluation was normal. Laboratorial analyses showed mild anemia (hemoglobin 9.4 mg/dL), worsening kidney function (previous creatinine 1.4, after 1.81 mg/dL), high inflammatory markers (c-reactive protein 10 mg/dL, ferritin 775 mg/dL), and low LDH levels (98 UI/mL). Computed tomography showed generalized lymphadenopathies in mediastinal, mesenteric, iliac, and retroperitoneal chains, without liver or spleen alterations. Additionally, bilateral lung infiltrates were detected (Fig 2).

Sputum screening for tuberculosis using culture and GeneXpert MTB/RIF and anti-HIV antibodies were negative. Fungal direct microscopic examination of the sputum with 10% potassium hydroxide (KOH) and serum cryptococcal antigen (IMMY diagnostics, Norman, OK, USA) were negative as well. Serum antibody detection using double immunodiffusion (DID) against antigens of *Paracoccidioides* spp., *Histoplasma capsulatum*, and *Aspergillus* spp. also yielded negative results. The Clarus *Histoplasma* Galactomanan Enzyme Immunoassay (IMMY diagnostics) from the urine was positive (2.234 EIA Units). Then, the patient was promptly started on liposomal amphotericin B (AmbL) with a presumptive diagnosis of progressive disseminated histoplasmosis.

A week later, direct microscopic examination of a tissue fragment obtained from the skin lesions showed *Paracoccidioides* sp. yeast-like cells. In addition, fungal cultures from the skin fragment and the sputum also yielded *Paracoccidioides* sp., after a month of incubation. A nested-polymerase chain reaction [20] from the sputum was conducted, to aid in the exclusion of histoplasmosis diagnosis, yielding negative results.

Given the underlying immunosuppressive condition of the patient and the subacute progression of the clinical symptoms, with the presence of fever, joint pain, disseminated lymph node involvement, disseminated skin lesions, and need for hospitalization, we made a final diagnosis of acute PCM. The patient received AmbL (3 mg/kg/day) for 14 days with general clinical improvement. However, treatment was changed to itraconazole (ITZ) 200 mg/day due to worsening kidney function and hyperkalemia. He was discharged with ITZ, which was further replaced by co-trimoxazole due to drug interactions with tacrolimus. The patient was still undergoing PCM treatment, when he died due to a non-related PCM cause (Epstein-Barr virus associated diffuse large B-cell lymphoma affecting the central nervous system).

### Literature review

A comprehensive literature search was conducted to investigate previous studies that found cross-reactivity between *Histoplasma* urinary antigen assays in human patients with confirmed diagnosis of PCM, using the following terms “cross-reactivity Histoplasma antigen” OR “Histoplasma urinary antigen”. Additionally, the following descriptors “Paracoccidioides” AND “transplant” were submitted to the same database aiming to search for articles reporting human cases of PCM in transplant recipients.

Both searches were conducted at PUBMED database, regardless the publication’s date, and included papers in English. Reviews and non-human models were excluded. Additional human case reports and cases series missed in the initial search strategy were carefully searched from the reference lists of the articles initially retrieved.

## Results

In the literature review about *Histoplasma* urinary antigen assays, a total of 160 manuscripts were retrieved. Among them, twelve manuscripts about cross-reactivity between *Histoplasma capsulatum* urinary antigen assays in human patients with PCM, published between 1997 and 2023, were carefully selected [15,16,21–31]. Table 1 shows details of these articles, including the method used for antigen detection, the number of samples from patients with PCM tested, the number of urine samples from participants with PCM that were reactive in *Histoplasma* antigen detection, and the cutoff values used in the study. The included studies reported a total of 120 samples from patients with PCM that were tested for *Histoplasma* antigen, presenting an overall cross-reactivity of 62/120 (51.67%). We excluded reference [30] from this list because of non-specified cross-reactivity rate.

**Table 1:** Manuscripts retrieved in the literature about cross reactivity of *Histoplasma capsulatum* urinary antigens with *Paracoccidioides* spp.

Year	Method	PCM samples tested	Positive samples	Cut-off values	Reference number
1997	Solid-phase ELISA	9	8	Cutoff 1.0 U	[15]
1997	Inhibition ELISA	11	3	Cutoff 4.4 μg/mL	[23]
1997	Solid-phase ELISA	1	1	Cutoff 1.0 EU	[24]
	Solid-phase RIA	1	1	Cutoff 1.0 RU	
2000	Sandwich ELISA	9	8	Cutoff 1.0 EU	[22]
	Inhibition ELISA	9	4	Cutoff 0.01 μg/mL	
2007	Sandwich ELISA	1	1	Cutoff 1.0 U	[21]
2007	Sandwich ELISA	5	4	Cutoff 0.6 ng/mL	[16]
2009	Indirect ELISA	25	8	Cutoff 0.7 U	[25]
2014	Capture ELISA	32	8	Cutoff 0.84 ng/mL	[26]
2018	Capture ELISA	3	3	Cutoff 0.5 ng/mL	[27]
2021	MVD GM LFA	2	2	Qualitative test Cutoff 0.2 ng/mL	[28]
	MVD GM ELISA	2	2		
2021	MVD GM LFA	7	6	Qualitative test	[29]
2023	IMMY GM ELISA	3	3	Cutoff 0.2 ng/mL	[31]
**Total**		**120**	**62**		

EU = ELISA Units; ELISA **=** Enzyme Liked Immunosorbent Assay; GM = Galactomannan; MVD = MiraVista Diagnostics; LFA = lateral flow assay; RIA = Radioimmunoassay; RU = RIA Units.

With regards to the literature review on PCM occurring in SOT recipients, 15 articles were identified. Among them, five were reviews or animal studies, thus excluded from this work, totalizing 10 included papers [32–41]. Additional four articles were incorporated into the present review from the reference lists of the 10 articles previously included [42–45]. Among these 14 included papers, 12 reported individual cases of PCM after transplant [32–39,42–45]. Additionally, one retrospective study of necropsy findings in renal transplant recipients included four PCM cases [40]. Furthermore, one retrospective study performed on bronchoalveolar specimens collected from patients with pneumonia after lung transplant reporting one lung infection due to *Paracoccidioides* sp. [41]. Together, these studies document 17 patients. Table 2 summarizes the main data of the case herein reported as well as those from the previously published cases of PCM in transplant recipients retrieved in the literature search that the authors conducted.

**Table 2:** Manuscripts retrieved in the literature concerning paracoccidioidomycosis in solid organ transplant recipients with their main clinical and laboratorial findings. Details of the case reported in the present study are also provided.

Year	SOT	Symptoms onset	SMX-TMP prophylaxis	PCM Clinical Form	PCM Diagnosis	PCM Serology	Treatment	Outcome	Reference number
1984	Kidney	NA	NA	NA	NA	Positive	KTZ	Improved	[32]
1995	Kidney	5 years	NA	Chronic	DE (BAL)	Positive (CIE 1:4; CF 1:8)	AMB > KTZ	Death	[33]
1995	Kidney	NA	NA	NA	NA	NA	NA	One death in 4 PCM cases	[40]
2004	Kidney	14 years	No	Chronic	DE (sputum)	Positive (DID with 2 bands)	AMB > ITZ	Improved	[34]
2008	Lung	NA	NA	NA	NA	NA	NA	NA	[41]
2015	Kidney	3 years	No	Acute	HP (lymph node biopsy)	NA	SMX-TMP	Improved	[35]
2016	Kidney	1 year	No	Chronic	DE (BAL)	NA	ITZ > AMB	Death	[36]
2017	Kidney	2 days	Yes	Acute	DE (BAL)	Negative	SMX-TMP > AMB > ITZ	Improved	[37]
2017	Liver	2 years	No	Acute	HP (lymph node biopsy)	Positive (CIE 1:64)	SMX-TMP > SMX-TMP + AMB > SMX-TMP	Improved	[38]
2019	Liver	1 year	No	Acute	DE (abscess)	Negative	AMB > SMX-TMP	Improved	[39]
2021	Liver	1 year	No	Acute	HP (skin biopsy)	Positive (DID 1:8)	AMB > SMX-TMP	Improved	[42]
2021	Liver	1 year	No	Acute	HP (skin biopsy)	Negative	AMB > ITZ	Improved	[43]
2021	Kidney	5 years	No	Chronic	HP (trans bronchial biopsy	NA	AMB > ITZ	Improved	[44]
2022	Heart	NA	NA	Acute	NA	NA	VRZ > SMX-TMP	Improved	[45]
2024	Kidney	7 months	No	Acute	DE (skin biopsy) and cultures (skin biopsy and sputum)	Negative	AMB > ITZ > SMX-TMP	NA	**This work**

SOT = solid organ transplanted; NA = not available; DE = Direct Examination; HP = histopathology; BAL = Bronchoalveolar lavage; CIE = Counterimmunoelectrophoresis; CF = Complement Fixation; DID = Double Immunodiffusion; KTZ = ketoconazole; ITZ = itraconazole; AMB = amphotericin B; SMX-TMP = sulfamethoxazole-trimethoprim.

## Discussion

Low- and middle-income countries have been facing a double burden of communicable and non-communicable diseases, with increasing prevalence of chronic conditions leading to higher need for SOT, and persistently high incidence of infectious diseases [46]. A previous literature review of PCM in immunosuppressed patients [6] identified 10 cases of PCM in SOT recipients during 30 years of the studied period, up to 2017 [34–38,40,41]. Since then, we detected five additional cases of PCM in SOT [39,42–45]. Although PCM after SOT is rare, this increasing number of published case reports could indicate a potential role of *Paracoccidioides* spp. as an emerging fungal pathogen causing acute and atypical manifestations in these patients.

In our case, the patient had no previous report of epidemiological risk activities for PCM infection. Therefore, we cannot estimate when fungal infection occurred. Although the highest incidence of infections in SOT recipients occurs during the first year after transplantation, most published cases of PCM in these patients report this occurrence after the first year. This may be probably due to the routine use of cotrimoxazole for *Pneumocystis jirovecii* prophylaxis during the first year after transplantation, an antimicrobial also effective against *Paracoccidioides spp*. [6]. As our patient was in his 5^th^ year after transplantation when PCM clinical symptoms first appeared, we envisage that it is more likely that the fungal exposure occurred not that long ago. Regardless the moment of infection, acute and chronic PCM are mostly classified based on the immunological response of the host and their resulting clinical findings. Due to the underlying immunosuppressive condition of our patient, the subacute clinical progression of the clinical symptoms with the presence of fever, joint pain, disseminated lymph node involvement, and disseminated skin lesions, this case was further diagnosed with acute PCM [1,47]. Noteworthy, the classification of PCM clinical forms in immunocompromised hosts can be challenging due to coexisting features of both acute and chronic forms, which was previously described in people living with HIV/AIDS as mixed form [47].

PCM epidemiology is changing over the last decades due to rampant deforestation, mechanization in the rural environment, and human migration across the country [17]. Reports have indicated the emergence of acute PCM in urban areas, notably in Rio de Janeiro state, Brazil, the origin of the case presented here [18,48]. The PCM urbanization can represent a higher risk of infection for vulnerable populations [49,50]. In these cases, the clinical presentation may be atypical, leading to delay or misdiagnosis of this fungal disease [48]. This could mean an increased number of cases not identified due to the lack of routine surveillance or awareness from healthcare providers.

The laboratorial diagnosis of PCM in immunocompromised patients is particularly difficult. In the case herein presented, direct examination and fungal culture confirmed the PCM diagnosis. However, limitations of such methods are worth to mention. Microscopy is quick to perform, but has staff- and sample-dependent sensitivity [51,52]. Culture-based techniques and histopathology are resource and time-consuming and could lead to late diagnosis, with an impact on mortality [1,3].

Concerning the main techniques used for antibody detection, despite their good sensitivity and specificity, there is an increased incidence of false negative results in the context of the emergence of severe PCM disease and infection in immunosuppressed patients [1,6,51]. A single-center study revealed 84.2% positivity rate in DID tests among PCM patients living with HIV [49]. Nonetheless, the utility of serological tests in patients with other types of immunosuppression remains uncertain, due to the potential risk of false negative results in such context [6]. The results herein presented revealed 62.5% positivity in antibody-based immunologic tests for *Paracoccidioides* in the eight previously reported patients with PCM and SOT, suggesting that the efficacy of immunological tests for PCM in transplant-recipient may be lower than that observed in HIV-immunosuppressed patients. Besides, a high divergence on the in-house immunological methods among regional laboratories has been reported in Brazil, which is critical considering the different antigenic expression of the newly described *Paracoccidioides* species [53,54], and could potentially complicate the application of these methods in the clinical management of such patients.

In SOT recipients, early diagnosis of fungal infections is crucial for better outcomes, because immunosuppression can lead to rapid progression and worse clinical pictures. Some assays have been proposed as important tools for the rapid diagnosis of endemic mycoses such as cryptococcosis and histoplasmosis. A lateral-flow-based *Histoplasma* antigen detection in urine samples has been proposed as a novel and efficient diagnostic tool for the rapid diagnosis of disseminated histoplasmosis, due to its good performance [28]. However, the galactomannan antigen used in this assay is not specific for *Histoplasma* and cross-reactivity with other endemic fungi can limit its specificity notably in tropical countries where many endemic mycoses can present epidemiological overlap [15,16,21–31]. Particularly, *Paracoccidioides* spp. present galactomannan in their cell-wall [55,56], sharing chemical and immunological characteristics with the *H*. *capsulatum* galactomannan [57], explaining, at least partially, the notable cross-reactivity determined in the current review.

The development of quick, easy-to-perform, and low cost tests presenting increased sensitivity and good specificity for the diagnosis of PCM in immunosuppressed patients is, therefore, imperative. Rapid tests based on antigen or another biomarker detection sharing a high overlap of the diagnostic target on the major agents of endemic mycoses is a high concern in settings where several endemic mycoses occur. Fortunately, severe presentations of endemic mycoses usually are successfully treated with amphotericin B, a broad-spectrum antifungal drug [58]. Therefore, the initial suspicion of disseminated histoplasmosis in our case did not change the first therapeutic choice that would be prescribed to treat severe acute PCM.

Alternatively, molecular methods are fast and safer alternatives to diagnose PCM directly from clinical samples, but they usually require high-cost infrastructures and consumables, and then they are not widely available in PCM endemic areas [59].

The study herein conducted calls attention to the emerging role of endemic mycoses, specifically PCM, as important causes of invasive fungal infection in SOT recipients. Better surveillance strategies as well as more rapid, accurate, and specific diagnostic tools are urgently needed to improve the diagnosis of opportunistic PCM in this vulnerable population.

**Key learning points**

1. Healthcare providers must be aware of the increasing occurrence of PCM in urban areas, affecting immunosuppressed patients such as SOT recipients.
2. PCM must be included in the differential diagnosis of other opportunistic mycoses in patients with underlying immunosuppressive conditions.
3. False negative serological results can occur in the context of immunosuppression and the galactomannan urinary antigen developed to diagnose histoplasmosis can cross react with PCM, which may represent a concern in countries where both mycoses overlap.
4. It is imperative to develop new rapid tests as those based on specific antigenic detection to improve the diagnosis of PCM in immunosuppressed patients and to refine current existing tests for histoplasmosis diagnosis, to reduce the occurrence of false-positive results.
